# Perirhinal Contributions to Human Visual Perception

**DOI:** 10.1016/j.cub.2007.07.066

**Published:** 2007-09-04

**Authors:** Joseph T. Devlin, Cathy J. Price

**Affiliations:** 1 Department of Psychology, University College London, London, WC1E 6BT, United Kingdom; 2 Wellcome Trust Centre for NeuroImaging, University College London, London, WC1N 3BG, United Kingdom

**Keywords:** SYSNEURO

## Abstract

Medial temporal lobe (MTL) structures including the hippocampus, entorhinal cortex, and perirhinal cortex are thought to be part of a unitary system dedicated to memory [1, 2], although recent studies suggest that at least one component—perirhinal cortex—might also contribute to perceptual processing [3–6]. To date, the strongest evidence for this comes from animal lesion studies [7–14]. In contrast, the findings from human patients with naturally occurring MTL lesions are less clear and suggest a possible functional difference between species [15–20]. Here, both these issues were addressed with functional neuroimaging in healthy volunteers performing a perceptual discrimination task originally developed for monkeys [7]. This revealed perirhinal activation when the task required the integration of visual features into a view-invariant representation but not when it could be accomplished on the basis of simple features (e.g., color and shape). This activation pattern matched lateral inferotemporal regions classically associated with visual processing but differed from entorhinal cortex associated with memory encoding. The results demonstrate a specific role for the perirhinal cortex in visual perception and establish a functional homology for perirhinal cortex between species, although we propose that in humans, the region contributes to a wider behavioral repertoire including mnemonic, perceptual, and linguistic processes.

## Results and Discussion

To investigate whether human perirhinal cortex contributes to perception in a manner homologous to that seen in macaques, we conducted a functional neuroimaging study with healthy volunteers performing a visual discrimination task used previously in both animal [7] and human patient studies [15, 16, 18]. The task was designed to engage perceptual, but not mnemonic, processes by presenting participants with a visual array of four images and forcing them to choose the odd one out. Because the duration of the stimuli on the screen was longer than the response time, the task did not require stimuli to be encoded into memory. In addition, unlike monkeys, who require extensive training to learn the task, humans are able to perform the task immediately, effectively eliminating any significant contributions of learning [21]. Both proponents and opponents of the claim that human perirhinal cortex contributes to perception have used this task as a test of perceptual processing with minimal mnemonic demands [15, 16, 18, 19].

**Figure 1:**
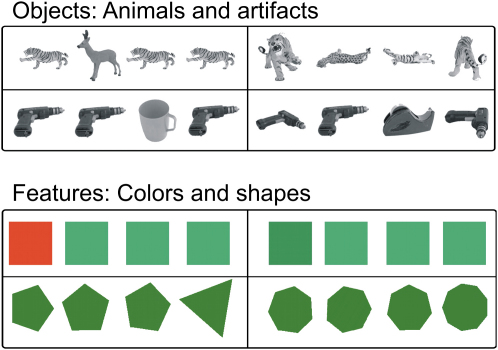
Example Stimuli Used in the Experiment, Divided into the Four Main Experimental Conditions Easy (left column) and difficult (right column) trials are shown for object (top rows) and feature (bottom rows) stimuli. It is worth noting that the color reproduction on the page may not exactly correspond to that used in the experiment because of slight differences in the methods used to represent color.

In the current study, stimuli came from four different conditions drawn from a 2 × 2 factorial design that crossed the stimulus type (object versus feature) with the processing level (difficult versus easy). Sample stimuli arrays are illustrated in Figure 1. Object stimuli were digital pictures of real 3D animals or artifacts, whereas feature stimuli were either patches of color or simple geometric shapes. In easy trials, the different item stood out and was readily identifiable, whereas the difficult trials required greater effort. Even so, difficult feature trials could be performed on the basis of a single feature (either color or shape), whereas difficult objects required identifying that three different images represented a single object shown from different views (see the Supplemental Experimental Procedures available online). Consequently, the ability to correctly perform these trials relied on integrating multiple visual features to identify the object at an abstract (i.e., view invariant) level. Thus, the critical distinction was that only the difficult object condition required objects to be identified at an abstract level and therefore was expected to activate perirhinal cortex. All other conditions, regardless of difficulty, could be performed by feature matching, which would be expected to engage ventral visual stream structures rather than perirhinal cortex. If the perirhinal cortex contributes to perception by integrating multiple features into a more abstract, object-level representation, then difficult object discriminations would be expected to selectively activate this region, whereas simple feature matching will not. This would suggest a functional homology between species. In contrast, if the perirhinal cortex differs between human and nonhuman primates, we would expect these perceptual discriminations to solely activate visual areas such as lateral inferotemporal lobe structures thought to be homologous to TE and TEO in macaques.

**Figure 2:**
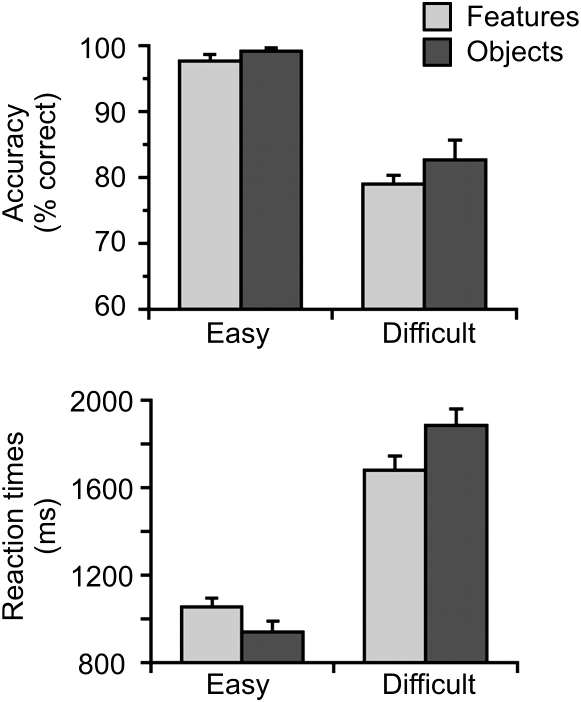
Behavioral Results The top panel displays participants' accuracy scores, and the bottom displays their reaction times for both feature (light gray) and object (dark gray) conditions. In both cases, the difference between easy and difficult trials was highly significant, and for RTs, but not accuracy, there was also a significant interaction. Error bars represent standard error of the mean.

The behavioral results are shown in Figure 2. The overall accuracy was high (88.1%) and strongly affected by the processing level, with significantly greater accuracy for easy (98.4%) than for difficult trials (80.8%; F_1,10_ = 153.7, p < 0.001). There was no significant main effect of the stimulus type, nor was there a significant interaction (both F_1,10_ < 2.0, not significant [n.s.]). The lack of an interaction is important because it means that any activation that is specific to difficult objects is not confounded by differences in accuracy between the two difficult conditions [22]. A similar pattern was observed in the reaction times (RTs) with a significant effect of the processing level (F_1,10_ = 583.7, p < 0.001) but not the stimulus type (F_1,10_ = 0.6, p = 0.45). There was, however, a significant crossover interaction (F_1,10_ = 11.7, p = 0.007), indicating that for easy trials, objects were faster than features, but that this pattern reversed for difficult trials.

**Figure 3:**
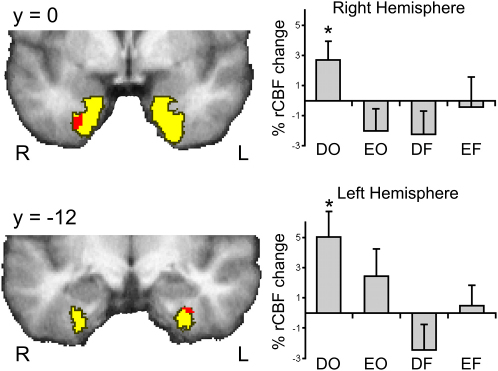
Perirhinal Activation for the Interaction between Stimulus Type and Processing Level The left-hand panels show that the activated voxels (red) within the perirhinal region of interest (yellow) defined as those voxels with a 50% or greater likelihood of being perirhinal cortex from the probabilistic map (Supplement Data). Bar plots in the right panels show the percent regional cerebral blood flow (%rCBF) change for each condition relative to baseline in the right and left perirhinal cortex. Error bars represent standard error of the mean, and significant differences from baseline are indicated with an asterisk (“^∗^”). Only the difficult object discriminations led to significant activation relative to baseline in perirhinal cortex. The following abbreviations are used: difficult objects (DO), easy objects (EO), difficult features (DF), easy features (EF).

The functional images were analyzed so that it could be determined whether difficult object decisions activated perirhinal cortex more than the other three conditions—in other words, whether there was an interaction between the stimulus type and processing level. Perirhinal cortex was anatomically defined on each participant's T1-weighted scan and then combined into a group-based probabilistic atlas (see the Supplemental Data). Within this region of interest, the interaction was significant at p < 0.05 after correcting for multiple comparisons at 28, 0, −42 (Z = 3.45, 36 voxels) in the right and at −34, −12, −34 (Z = 3.22, 20 voxels) in the left (Figure 3). The bar plots in Figure 3 illustrate the percent regional cerebral blood flow (rCBF) change per condition relative to the mean of the two baseline conditions within the activated regions of perirhinal cortex. The difficult object condition was the only one to activate perirhinal cortex above baseline (right: t_11_ = 3.09, p = 0.010; left: t_11_ = 2.35, p = 0.047); no other condition was significantly different from baseline (active or deactive, all n.s.). In addition, all of the activated voxels in the right hemisphere had a 50% or greater likelihood of being perirhinal cortex as defined by our anatomically based probabilistic mask. In the left hemisphere, 16 our of 20 voxels were within the 50% boundary, and the remaining 4 out of 20 had a 40% likelihood of being perirhinal cortex. In other words, these activations were located entirely within anatomically defined perirhinal cortex and therefore could not be due to activation spreading from the more medial entorhinal cortex nor lateral inferotemporal regions.

Because both the reaction times and the rCBF changes in perirhinal cortex exhibited a crossover interaction, with longer responses and larger rCBF changes for difficult relative to easy objects, a second analysis of the imaging data was conducted that included RTs as a covariate so that it could be determined whether the perirhinal findings could be explained either as increased working memory demands [23, 24] or in terms of a generalized difficulty effect. The results showed that (1) the perirhinal activations were essentially unchanged after RTs were modeled out (both peak Z scores changed by less than 0.1) and that (2) activation was not predicted by RTs in either the right (Z = 0.81) or left (Z = 0.89) perirhinal cortex. These findings demonstrate that perceptual decisions performed on difficult objects engaged bilateral perirhinal cortex. The activations could not be explained as a linear function of overall task difficulty because reaction times were not a significant predictor of activation levels. In addition, easy object, difficult feature, and easy feature discriminations did not significantly activate perirhinal cortex relative to baseline, suggesting that tasks that can be performed on the basis of feature matching alone do not require perirhinal cortex. The question then becomes this: Is this activation evidence for perirhinal involvement in perception, or could it be explained in terms of mnemonic processes?

**Figure 4:**
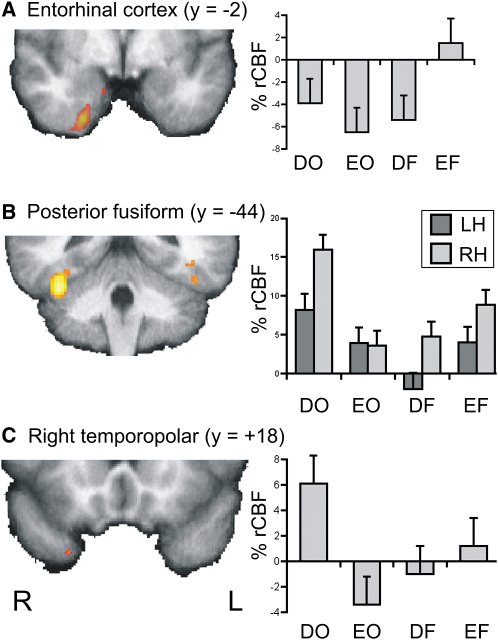
Entorhinal and Inferotemporal Activation Activation for the interaction between stimulus type and processing level in right entorhinal cortex (A), bilateral posterior fusiform gyrus (B), and right temporopolar cortex (C). In the left column, activations are shown on coronal slices through the group mean structural image with a color scale ranging from Z = 1.66 (red) to Z = 3.0 (yellow). In the right column, bar plots show the percent rCBF relative to baseline per condition for activation in the left (dark gray) and right (light gray) hemispheres. See Figure 3 for abbreviations.

Although the visual discrimination task did not *require* mnemonic processing, it is nonetheless possible that the difficult object condition increased demands on memory, leading to higher perirhinal activation. For instance, if stimuli were implicitly encoded into memory, then the four unique object views in the difficult condition would be more demanding than two views in the easy condition. Alternately, participants might have adopted a strategy for difficult object trials, in which they recognized each image in the array as an exemplar of a stored prototype (e.g., tiger), thus drawing on declarative memory. In contrast, all the visual information needed to solve the task was present in the display for the other three conditions without reference to stored memories. Consequently, a mnemonic explanation of our perirhinal activation can not be completely ruled out. Nevertheless, it is instructive to compare the patterns of activation in the perirhinal and entorhinal cortices because the latter is considered an essential component of the declarative memory system [25]. Contrary to the response in the perirhinal cortex, there was no significant stimulus type × processing level interaction in the entorhinal cortex, even when the threshold was lowered to p < 0.001 uncorrected. Moreover, if we lowered the threshold to p < 0.05 uncorrected, a small unilateral activation in the entorhinal cortex was revealed at 14, −2, −26 (Z = 1.96, 6 voxels), but unlike the perirhinal activations, the only condition showing activation above baseline was easy features; all other conditions showed deactivations (Figure 4A). In other words, within entorhinal cortex, the activation was both weak and driven primarily by the other tail of the interaction, suggesting that activation in perirhinal and entorhinal cortices was not the product of a single, common process and was not consistent with an explanation of perirhinal activation in terms of encoding into or retrieving from declarative memory.

In contrast, lateral inferotemporal regions classically associated with higher-order visual perception showed similar patterns of activation to perirhinal cortex (Figures 4B and 4C). Posteriorally, strong activation was observed in the right fusiform gyrus at 40, −44, −24 (Z = 4.96, 184 voxels) with a corresponding, but slightly weaker, activation in the left hemisphere at −48, −48, −22 (Z = 3.00, 30 voxels). Relative to baseline, all conditions were significantly activated in the right fusiform and all but one in the left. In both hemispheres, the strongest activation was for difficult objects. More anteriorally, there was also activation present in right temporopolar cortex at 30, 18, −44 (Z = 2.45, 10 voxels), as shown in Figure 4C. Here, only the object conditions were above baseline with greater activation for difficult relative to easy objects. This pattern of results is consistent with the existence of increasingly complex neuronal receptive fields as visual information progresses rostrally through inferotemporal cortex [26, 27]. Moreover, it suggests that tasks that require combining visual information into a more abstract representation of the stimuli than was present in the visual image engage a distributed set of temporal lobe regions, including perirhinal cortex. In other words, in this experiment, perirhinal activation patterned with regions traditionally linked with visual perception rather than regions such as entorhinal cortex associated with memory.

Like previous studies of patients with naturally occurring medial temporal lobe (MTL) lesions [18–20, 28], we observed functional differences between perirhinal and entorhinal cortices, suggesting that the widely accepted view of a functionally unitary MTL memory system including the hippocampus, perirhinal cortex, and entorhinal cortex, in which all of the structures contribute more or less equally to declarative memory, might be incorrect. Instead, these regions appear to have dissociable functions, at least for certain tasks or stimuli [18–20, 29, 30]. Moreover, the current findings go beyond previous lesion studies by identifying specific areas of perirhinal cortex involved in higher-order visual perception. They also go beyond that of a previous functional imaging study that observed perirhinal activation when object and spatial discriminations were compared [31]. Specifically, the response properties of our perirhinal activation are consistent with the integration of visual features into an abstract representation, and this allows us to draw an important functional homology between humans and monkeys.

Given these results, why have some studies found perceptual deficits in human patients with bilateral perirhinal lesions [18–20], although others have not [15–17]? One possibility is that some of these patients might have retained residual perirhinal function. In three herpes simplex encephalitis patients, for example, T2-weighted magnetic resonance (MR) imaging revealed extensive bilateral medial temporal damage apparently affecting all of perirhinal cortex [16]. Although structural MRI can identify damaged tissue, it cannot rule out the possibility that within the lesion there remains some functionally viable tissue capable of contributing to a task [32]. Therefore, without functional imaging data on these patients, it is impossible to rule out this explanation. Another possibility is that patients with good accuracy despite focal lesions might be able to adopt an atypical strategy for solving the task that does not rely on perirhinal function—one that if linguistically based, would not be available to monkeys. Reaction-time data from patients with perirhinal lesions would help to determine whether their performance was entirely within the normal range. Finally, it is worth noting that inconsistencies in the results from patients could result from the choice of stimuli and the performance levels of healthy controls [31, 33]. In short, additional data are required to establish the effect of perirhinal lesions in human patients.

Our proposal that parts of perirhinal cortex are involved in representing conjunctions of features [3, 12] appears consistent with a range of human neuroimaging findings. For instance, perirhinal cortex is activated by difficult same-different judgments on pictures of human faces [34] and by successful integration of visual and tactile information [35], both of which require integrating featural information. Moreover, perirhinal lesions in macaques impair performance on similar face discriminations [7] and for visual-tactile associations [36]. Interestingly, perirhinal activation has also been reported for purely linguistic stimuli such as auditory [37] or written [38–40] sentences. The activation, however, appears to be driven by word, rather than sentence, processing because both single words and sentences activate the region to the same extent [39, 40]. If true, then human perirhinal cortex is functionally homologous with macaques [4], although in humans it might contribute to a wider behavioral repertoire including mnemonic, perceptual, *and linguistic* processes.

By this account, the perirhinal cortex need not store complex feature conjunctions per se. Instead, by virtue of its widespread and polymodal afferents [41], it temporarily instantiates these patterns in its neuronal firing to dynamically link otherwise disparate information. Consequently, an important next step is to elucidate its functional interactions. In the macaque, perirhinal cortex receives widespread, multimodal input and projects to a diverse set of neocortical, allocortical, and limbic association areas. Recent advances in diffusion-weighted imaging now make it possible to investigate these pathways noninvasively in humans to determine whether the anatomical connectivity profile is conserved across species. In addition, functional connectivity studies should help to clarify whether perirhinal cortex interacts differently with other regions depending on the task. For instance, one might predict stronger functional links with lateral inferotemporal regions during visual object discrimination than during delayed matching-to-sample tasks, which presumably have stronger interactions with prefrontal cortex. To this end, the probabilistic atlas of human perirhinal cortex presented in the Supplemental Data will hopefully assist others by facilitating the reliable identification of perirhinal cortex as distinct from other components of the medial temporal lobe and more rostral temporopolar cortex.

## Experimental Procedures

Twelve healthy volunteers performed a visual discrimination task modified from [7] for use with humans during positron emission tomography (PET) scanning. On each trial, four visual images were presented horizontally, and the volunteer indicated the image that was different from the other three. Multiple views of the same objects were used to generate difficult object trials, whereas the same view of an object was repeated three times in the easy object trials. Consequently, correct responses during the difficult trials relied on integrating multiple visual features to identify the object at an abstract (i.e., view invariant) level, whereas easy trials could be performed via visual feature matching. Feature stimuli were either patches of color or filled green polygons. Difficult color decisions consisted of three identical color patches and a slight variant, whereas easy color decisions used a different color for the nonmatching item. Shape decisions used three identical, but rotated, polygons and a fourth with either two fewer or greater sides. Difficult trials involved six versus eight, seven versus nine, or eight versus ten sides, whereas easy trials involved three versus five or four versus six sides. In all feature conditions, trials could be distinguished solely in terms of a single visual feature, namely color or shape. The baseline involved fixation and a button press. See the Supplemental Data for more details.

## Supplemental Data

Document S1. Experimental Procedures and Two Figures

## References

[1] Squire L.R., Stark C.E., Clark R.E. (2004). The medial temporal lobe. Annu. Rev. Neurosci..

[2] Squire L.R., Bayley P.J. (2007). The neuroscience of remote memory. Curr. Opin. Neurobiol..

[3] Murray E.A., Bussey T.J. (1999). Perceptual-mnemonic functions of the perirhinal cortex. Trends Cogn. Sci..

[4] Buckley M.J., Gaffan D. (2006). Perirhinal cortical contributions to object perception. Trends Cogn. Sci..

[5] Bussey T.J., Saksida L.M. (2005). Object memory and perception in the medial temporal lobe: an alternative approach. Curr. Opin. Neurobiol..

[6] Murray E.A., Richmond B.J. (2001). Role of preirhinal cortex in object perception, memory, and associations. Current Opinions in Neurobiology.

[7] Buckley M.J., Booth M.C., Rolls E.T., Gaffan D. (2001). Selective perceptual impairments after perirhinal cortex ablation. J. Neurosci..

[8] Bussey T.J., Saksida L.M., Murray E.A. (2003). Impairments in visual discrimination after perirhinal cortex lesions: testing ‘declarative’ vs. ‘perceptual-mnemonic’ views of perirhinal cortex function. Eur. J. Neurosci..

[9] Buckley M.J., Gaffan D. (1998). Learning and transfer of object-reward associations and the role of the perirhinal cortex. Behav. Neurosci..

[10] Buckley M.J., Gaffan D. (1997). Impairment of visual object-discrimination learning after perirhinal cortex ablation. Behav. Neurosci..

[11] Buckley M.J., Gaffan D. (1998). Perirhinal cortex ablation impairs visual object identification. J. Neurosci..

[12] Bussey T.J., Saksida L.M., Murray E.A. (2002). Perirhinal cortex resolves feature ambiguity in complex visual discriminations. Eur. J. Neurosci..

[13] Baxter M.G., Murray E.A. (2001). Impairments in visual discrimination learning and recognition memory produced by neurotoxic lesions of rhinal cortex in rhesus monkeys. Eur. J. Neurosci..

[14] Eacott M.J., Gaffan D., Murray E.A. (1994). Preserved recognition memory for small sets, and impaired stimulus identification for large sets, following rhinal cortex ablations in monkeys. Eur. J. Neurosci..

[15] Levy D.A., Shrager Y., Squire L.R. (2005). Intact visual discrimination of complex and feature-ambiguous stimuli in the absence of perirhinal cortex. Learn. Mem..

[16] Stark C.E., Squire L.R. (2000). Intact visual perceptual discrimination in humans in the absence of perirhinal cortex. Learn. Mem..

[17] Shrager Y., Gold J.J., Hopkins R.O., Squire L.R. (2006). Intact visual perception in memory-impaired patients with medial temporal lobe lesions. J. Neurosci..

[18] Lee A.C., Buckley M.J., Gaffan D., Emery T., Hodges J.R., Graham K.S. (2006). Differentiating the roles of the hippocampus and perirhinal cortex in processes beyond long-term declarative memory: a double dissociation in dementia. J. Neurosci..

[19] Barense M.D., Bussey T.J., Lee A.C., Rogers T.T., Davies R.R., Saksida L.M., Murray E.A., Graham K.S. (2005). Functional specialization in the human medial temporal lobe. J. Neurosci..

[20] Lee A.C., Bussey T.J., Murray E.A., Saksida L.M., Epstein R.A., Kapur N., Hodges J.R., Graham K.S. (2005). Perceptual deficits in amnesia: Challenging the medial temporal lobe ‘mnemonic’ view. Neuropsychologia.

[21] Hampton R.R. (2005). Monkey perirhinal cortex is critical for visual memory, but not for visual perception: Reexamination of the behavioural evidence from monkeys. Q. J. Exp. Psychol. B.

[22] Murphy K., Garavan H. (2004). Artifactual fMRI group and condition differences driven by performance confounds. Neuroimage.

[23] Ranganath C. (2006). Working memory for visual objects: Complementary roles of inferior temporal, medial temporal, and prefrontal cortex. Neuroscience.

[24] Lehky S.R., Tanaka K. (2007). Enhancement of object representations in primate perirhinal cortex during a visual working-memory task. J. Neurophysiol..

[25] Squire L.R. (2004). Memory systems of the brain: a brief history and current perspective. Neurobiol. Learn. Mem..

[26] Tanaka K. (1993). Neuronal mechanisms of object recognition. Science.

[27] Ungerleider L.G., Mishkin M., Ingles J.D., Goodale M.A., Mansfield R.J.W. (1982). Two cortical visual systems. Analysis of Visual Behaviour.

[28] Taylor K.J., Henson R.N., Graham K.S. (2007). Recognition memory for faces and scenes in amnesia: Dissociable roles of medial temporal lobe structures. Neuropsychologia.

[29] Murray E.A., Mishkin M. (1998). Object recognition and location memory in monkeys with excitotoxic lesions of the amygdala and hippocampus. J. Neurosci..

[30] Winters B.D., Forwood S.E., Cowell R.A., Saksida L.M., Bussey T.J. (2004). Double dissociation between the effects of peri-postrhinal cortex and hippocampal lesions on tests of object recognition and spatial memory: Heterogeneity of function within the temporal lobe. J. Neurosci..

[31] Lee A.C., Bandelow S., Schwarzbauer C., Henson R.N., Graham K.S. (2006). Perirhinal cortex activity during visual object discrimination: an event-related fMRI study. Neuroimage.

[32] Warburton E., Price C.J., Swinburn K., Wise R.J. (1999). Mechanisms of recovery from aphasia: Evidence from positron emission tomography studies. J. Neurol. Neurosurg. Psychiatry.

[33] Lee A.C., Barense M.D., Graham K.S. (2005). The contribution of the human medial temporal lobe to perception: Bridging the gap between animal and human studies. Q. J. Exp. Psychol. B.

[34] Gorno-Tempini M.L., Price C.J., Josephs O., Vandenberghe R., Cappa S.F., Kapur N., Frackowiak R.S.J. (1998). The neural systems sustaining face and proper-name processing. Brain.

[35] Holdstock J.S., Hocking J., Notley P.R., Price C.J. (2006). An fMRI investigation of visual and tactile information from novel objects. Neuroimage.

[36] Goulet S., Murray E.A. (2001). Neural substrates of crossmodal association memory in monkeys: the amygdala versus the anterior rhinal cortex. Behav. Neurosci..

[37] Cardillo E.R., Aydelott J., Matthews P.M., Devlin J.T. (2004). Left inferior prefrontal cortex activity reflects inhibitory rather than facilitatory priming. J. Cogn. Neurosci..

[38] McCarthy G., Nobre A.C., Bentin S., Spencer D.D. (1995). Language-related field potentials in the anterior-medial temporal lobe: I. Intracranial distribution and neural generators. J. Neurosci..

[39] Nobre A.C., McCarthy G. (1995). Language-related field potentials in the anterior-medial temporal lobe: II. Effects of word type and semantic priming. J. Neurosci..

[40] Vandenberghe R., Nobre A.C., Price C.J. (2002). The response of left temporal cortex to sentences. J. Cogn. Neurosci..

[41] Suzuki W.A., Amaral D.G. (1994). Perirhinal and parahippocampal cortices of the macaque monkey: Cortical afferents. J. Comp. Neurol..

