# Supplementary material for: Perirhinal Contributions to Human Visual Perception
Source: Curr Biol. 2007 Sep 4;17(17):1484–8. doi: 10.1016/j.cub.2007.07.066 (PMC1971135; doi:10.1016/j.cub.2007.07.066)
Supplement: Document S1. Experimental Procedures and Two Figures [file mmc1.pdf]

Supplemental Data

Perirhinal Contributions to Human Visual Perception

Joseph T. Devlin and Cathy J. Price

Supplemental Experimental Procedures

Subjects

Twelve healthy, native British-speaking volunteers (11M, 1F) participated in this study. Ages ranged from 18 to 65 years (mean = 44), and all were right handed according to self report. All were medically screened so anyone with a history of neurological or psychiatric disorder could be avoided. Participants were then briefed on scanner safety and gave written consent before taking part. Ethical approval was granted by the Medical Ethics Committee for the Institute of Neurology, and the paradigm conforms with the guidelines established by the Administration of Radioactive Substances Advisory Committee of the Department of Health, UK.

Object stimuli were grayscale digital pictures of real-world 3D items that were either animals or artifacts. Feature stimuli were either patches of color or filled green polygons. For the color trials, ten different base colors were used, and easy trials consisted of three identical patches of one base color (e.g., dark green) and a different base color (e.g., red). Difficult color trials, on the other hand, used a single base color, but the odd item was a slight variant of that color. So in all cases, the odd item could be distinguished solely in terms of a single feature (i.e., color), but the difficulty of the discrimination varied. For the polygon trials, all of the polygons had an equal surface area, but the number of sides ranged from three to ten. In addition, the polygons were rotated within plane around a central axis by 0°–80° in 10° increments. Easy trials used polygons with three to six sides, whereas difficult trials used polygons with six to ten sides. In all cases, the two differently shaped polygons in each trial had either an odd or even number of sides. This ensured that difference judgments could not be based purely on differences in parallel sides. Like color trials, the determination of the odd polygon was based solely on a single visual feature, namely shape.

Note that individual stimuli were repeated both within and between scans (e.g., the drill in difficult and easy objects), but the number of repeated stimuli was the same for difficult and easy conditions. Although these repetitions might involve a form of memory retrieval if participants recognize a stimulus as repeated, the number of such occurrences were balanced between conditions. The complete ruling out of the potential effects of incidental recognition, however, would require the use of trial unique stimuli [S1, S2].

Two additional baseline conditions were also included in the experiment. In one, participants fixated on a centrally presented cross for 90 s, whereas in the other, they were asked to indicate with a button press when the screen briefly flashed from white to black. This partially controlled for sustained visual attention and the manual action of button presses present in the visual discrimination task. Thus, there were a total of ten scans, two per condition (i.e., two baselines, two difficult objects, two easy objects, etc.) with the order counterbalanced across participants.

Imaging

Subjects were scanned at the Wellcome Trust Centre for NeuroImaging where they first had a high-resolution T1-weighted MRI scan (3D Modified Driven Equilibrium Fourier Transform [MDEFT] sequence [TR/TE/TI = 12.24 ms/3.56 ms/ 530 ms]). One hundred and seventy six sagittal partitions were acquired with an image matrix of 256 × 224 mm, yielding a final resolution of 1 mm^3. These were used for the registration of activation to anatomy and for the anatomical identification of perirhinal cortex in individuals. Because of time constraints, one participant did not receive a structural scan.

Given the proximity of perirhinal cortex to the air-tissue interfaces of the sinuses, we chose to use positron emission tomography (PET) rather than functional magnetic resonance imaging (fMRI) for our functional imaging. In fMRI, macroscopic magnetic susceptibility artifacts make the imaging of the anterior medial temporal lobes difficult because of both signal dropout and image distortion [S3]. Dropout in the medial anterior temporal poles seriously degrades the blood-oxygen-level-dependent (BOLD) signal from perirhinal cortex [S4], and image distortion can lead to significant spatial shifts in activation that further reduce the anatomical accuracy of the localization procedure. It is possible to collect additional data such as field maps to exclude participants with significant artifacts in this region [S5], but standard data-acquisition methods can be problematic. For instance, one such study found "perirhinal" activation posterior to anatomically defined perirhinal cortex [S6] (compare activation in their Figure 1 to the anatomically defined probabilistic map of perirhinal cortex in our Figure S2). Although it is certainly possible that the underlying BOLD signal was generated within perirhinal cortex, the image distortion present in most fMRI studies makes this difficult to determine with confidence.

Consequently, we used PET instead, which offers similar spatial resolution without the twin costs of dropout and distortion that severely affect anterior medial temporal lobe structures. Although early PET studies had limited spatial resolution (on the order of 1 cm), modern scanners acquire isotropic 2 mm voxels comparable to typical fMRI data (i.e., isotropic 3 mm voxels). So PET provides roughly equivalent spatial accuracy for identifying peak activations [S7], although its ability to distinguish neighboring peaks might be reduced relative to fMRI. In the current study, participants were scanned on an ECAT EXACT HR+ (model 962) PET scanner (Siemens/CTI [Knoxville, TN]) with collimating septa retracted. They received a 20 s intravenous bolus of $H_2^{15}O$ at a concentration

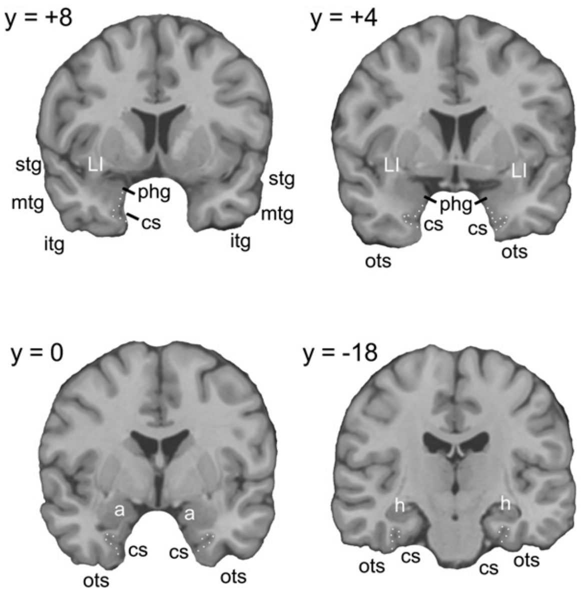

Figure S1. Anatomical Landmarks for Identifying Perirhinal Cortex

The top two panels illustrate the level of the limen insulae (LI), where perirhinal cortex extends from the half-way point along the parahippocampal gyrus (phg) to the lateral bank of the collateral sulcus (cs). The bottom two panels show more caudal planes through the amygdala (a) and hippocampus (h), respectively. Here, perirhinal cortex (indicated with white dots) extends from the midpoint on the medial wall of the collateral sulcus to the edge of the lateral wall. Above each panel is the y coordinate in standard (i.e., MNI) space of the coronal section. Note that in this brain, the rostral tip of the collateral sulcus appears at the level of the limen insulae, so there is no perirhinal cortex located more anteriorly. This was the most common pattern in our participants. The following abbreviations are used: inferior temporal gyrus (itg), middle temporal gyrus (mtg), occipitotemporal sulcus (ots), and superior temporal gyrus (stg).

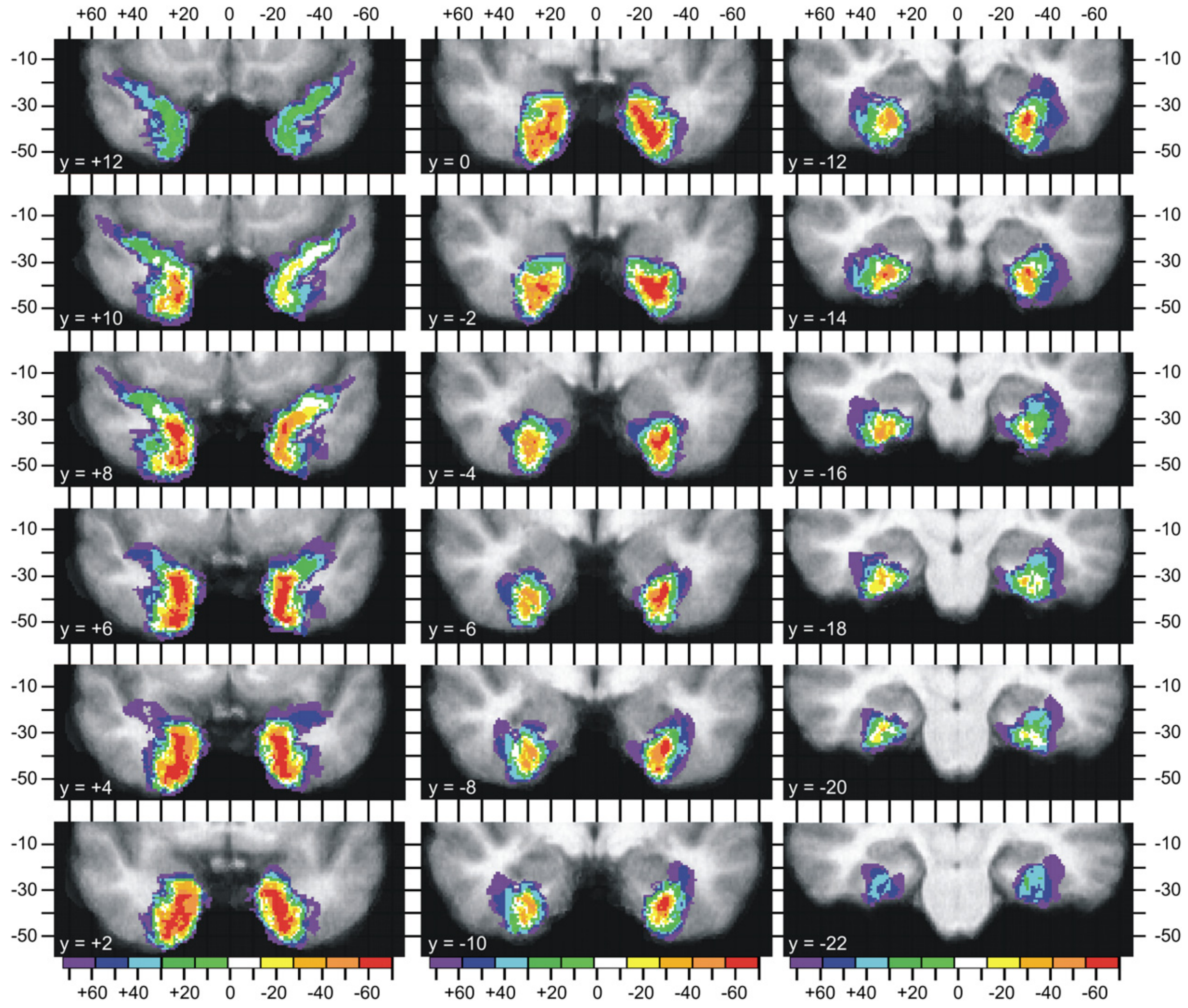

Figure S2. Probabilistic Map of Perirhinal Cortex in Standard Space Shown on the Mean Structural Scan

Individual voxels are color coded to indicate the likelihood of being perirhinal cortex in our participants. The colors, from purple to red, indicate degrees of likelihood (a 10% chance and a > 90% chance, respectively). These values are displayed on a series of coronal sections ranging from y = +12 to y = −22, shown in 2 mm increments. A reference grid of both x and z coordinates in 10 mm increments is provided.

of 55 Mbq/ml at a flow rate of 10 ml/min through a forearm cannula. The effective dose equivalent was < 5.0 mSv, as approved by the Administration of Radioactive Substances Advisory Committee of the Department of Health, UK. Correction for attenuation was made by a transmission scan with an exposed $^{68}Ge/^{68}Ga$ external source. Images were reconstructed by filtered back projection (Hanning filter, cutoff frequency 0.5 Hz) and produced voxels with a resolution of 2.05 × 2.05 × 2 mm. For our purposes, the critical factor was the ability to accurately localize activations in a predefined anatomical region, namely perirhinal cortex, in order to determine whether it was engaged when making difficult object discriminations and, if so, to identify the specific region(s) involved. Indeed, this spatial precision is clearly demonstrated in Figure 3, where activations were specific to perirhinal cortex without any evidence of spreading from either medial entorhinal or lateral inferotemporal regions.

Data Analysis

Reaction times (RTs) were recorded from the onset of the visual array. So that the effect of outliers in the RT data could be minimized, the median RT for correct responses was calculated per condition per subject for use in the statistical analyses. A hardware failure meant that for one participant, RTs were recorded in only three out of eight conditions; consequently, this person's behavioral data were not included in the analysis. Both RTs and accuracy were analyzed with repeated-measures analyses of variance (ANOVAs) with the stimulus type (object versus feature) and processing level (difficult versus easy) as independent factors. A second analysis included RTs per condition per participant as a covariate for all participants except the one whose RTs were not consistently recorded.

Next, the functional images were analyzed with SPM (Wellcome Trust Centre for NeuroImaging, http://www.fil.ion.ucl.ac.uk/spm). Images were first realigned to correct for small head motion [S8], and the mean image created by the realignment procedure was used for the determination of both the affine parameters and nonlinear warps for the transformation of the images onto the Montreal Neurological Institute (MNI) template [S8, S9]. These parameters were then applied to the functional images, maintaining the original spatial resolution of 2 mm isotropic voxels. Finally, each image was smoothed with an 8 mm at full-width half-maximum Gaussian filter. The SPM software used the general linear model and linear contrasts to compute the relevant statistics [S10]. Global cerebral blood flow counts were included as covariates of no interest. So that the selective activation for the difficult object condition could be identified, the interaction between the stimulus type and processing level was computed and inclusively masked at $p < 0.01$ uncorrected by the simple main effect of difficult > easy objects so that it could be ensured that activations were driven by the difference in object

stimuli, rather than the other tail of the interaction. All statistical comparisons were thresholded at $p < 0.05$ after correcting for multiple comparisons within the perirhinal region of interest, defined anatomically as those voxels that were classified as perirhinal cortex in at least 50% of the participants. This threshold was chosen as a fairly conservative method of ensuring the activation was in perirhinal cortex, given the variability present across individuals. Even more conservative thresholds (e.g., 8 out of 11 participants, ~75%) produced essentially identical results.

Anatomical Analyses

So that activation in perirhinal cortex could be reliably identified, our initial analysis of the imaging data began by identifying perirhinal cortex anatomically in the 11 participants for whom we had a high-resolution anatomical MRI scan. The T1-weighted image was first transformed into the MNI-152 standard space with an affine transformation and smoothly varying nonlinear warps [S9] while maintaining the initial resolution of 1 mm^3. Then perirhinal cortex was identified bilaterally on successive coronal slices according to the criteria of Insausti et al. [S11]. This procedure is illustrated in Figure S1. Along the entire rostrocaudal extent, the ventrolateral border of perirhinal cortex is determined by the depth of the collateral sulcus. For depths of 1 to 1.5 cm, the boundary of perirhinal cortex lies at the edge of the lateral bank of the collateral sulcus. In shallow cases (<1 cm depth), the border is located at the midpoint of the crest of the fusiform gyrus, whereas in deep cases (>1.5cm depth), the border is located at the midpoint of the lateral bank of the collateral sulcus. The dorsomedial border depends on the rostrocaudal level of the coronal section. Moving caudally from the temporal pole, the rostral-most border of perirhinal cortex appears at the anterior tip of the collateral sulcus, which can be a few millimeters anterior to the limen insulae in some subjects. At this level, perirhinal cortex is bounded dorsomedially by the fundus of the temporopolar sulcus, which separates it from the neocortex of the superior temporal gyrus, and here, perirhinal cortex can include one or two gyri of Schwalb. Slightly more caudally, at the level of the limen insulae, perirhinal cortex begins at the midpoint of the parahippocampal gyrus. At the level of the amygdala, the medial border of perirhinal cortex is located at the midpoint of the medial bank of the collateral sulcus, and this border remains the same to the most caudal extent of perirhinal cortex, approximately 2 mm posterior to the gyrus intralimbicus. Obviously, these borders are heuristic based on macroanatomic landmarks rather than definitive markers for the cytoarchitectonic boundaries of the region. They are, however, based on a large sample of cytoarchitectonic analyses (n = 49 postmortem brains) and specifically developed for the reliable identification of perirhinal cortex in high-resolution structural MRI images [S11].

Each anatomical mask was then restricted to only those voxels with at least a 20% probability of being gray matter according to an automated tissue segmentation tool [S12]. These were then combined to produce a probabilistic map of perirhinal cortex in standard space for use in the group analysis (Figure S2). There was considerable intersubject variability in the location of perirhinal cortex, both as a result of macroanatomic variability in the course of the collateral sulcus and also because of registration error, which is more pronounced at the edge of the brain. Only one-third of the total perirhinal volume (15,692/47,353 mm^3) was shared by at least 50% of the participants. This degree of anatomical variability is consistent with other neocortical structures defined either cytoarchitectonically [S13] or macroanatomically [S14] and highlights the difficulty in stating with certainty that an activation at a given coordinate is in perirhinal cortex. For individuals, Insausti et al.'s [S11] method remains the gold standard for localizing perirhinal cortex, whereas in group studies, it is necessary to adopt a probabilistic atlas in order to quantify the degree of certainty associated with perirhinal activation [S15, S16]. To this end, the map is available for download at http://joedevlin.psychol.ucl.ac.uk/perirhinal.php.

Supplemental References

S1. Lee, A.C., Bussey, T.J., Murray, E.A., Saksida, L.M., Epstein, R.A., Kapur, N., Hodges, J.R., and Graham, K.S. (2005). Perceptual deficits in amnesia: challenging the medial temporal lobe 'mnemonic' view. Neuropsychologia *43*, 1–11.

S2. Lee, A.C., Barense, M.D., and Graham, K.S. (2005). The contribution of the human medial temporal lobe to perception: Bridging the gap between animal and human studies. Q. J. Exp. Psychol. B *58*, 300–325.

S3. Jezzard, P., and Balaban, R. (1995). Correction for geometric distortion in echo planar images from B_o field variations. Magn. Reson. Med. *34*, 65–73.

S4. Devlin, J.T., Russell, R.P., Davis, M.H., Price, C.J., Wilson, J., Moss, H.E., Matthews, P.M., and Tyler, L.K. (2000). Susceptibility-induced loss of signal: Comparing PET and fMRI on a semantic task. Neuroimage *11*, 589–600.

S5. Lee, A.C., Bandelow, S., Schwarzbauer, C., Henson, R.N., and Graham, K.S. (2006). Perirhinal cortex activity during visual object discrimination: An event-related fMRI study. Neuroimage *33*, 362–373.

S6. Taylor, K.I., Moss, H.E., Stamatakis, E.A., and Tyler, L.K. (2006). Binding crossmodal object features in perirhinal cortex. Proc. Natl. Acad. Sci. USA *103*, 8239–8244.

S7. Mintun, M.A., Fox, P.T., and Raichle, M.E. (1989). A highly accurate method of localizing regions of neuronal activation in the human brain with positron emission tomography. J. Cereb. Blood Flow Metab. *9*, 96–103.

S8. Friston, K.J., Ashburner, J., Frith, C.D., Poline, J.-B., Heather, J.D., and Frackowiak, R.S.J. (1995). Spatial registration and normalization of images. Hum. Brain Mapp. *2*, 165–189.

S9. Ashburner, J., and Friston, K. (1999). Nonlinear spatial normalization using basis functions. Hum. Brain Mapp. *7*, 254–266.

S10. Friston, K.J., Frith, C.D., Turner, R., and Frackowiak, R.S. (1995). Characterizing evoked hemodynamics with fMRI. Neuroimage *2*, 157–165.

S11. Insausti, R., Juottonen, K., Soininen, H., Insausti, A.M., Partanen, K., Vainio, P., Laakso, M.P., and Pitkanen, A. (1998). MR volumetric analysis of the human entorhinal, perirhinal, and temporopolar cortices. AJNR Am. J. Neuroradiol. *19*, 659–671.

S12. Zhang, Y., Brady, M., and Smith, S. (2001). Segmentation of brain MR images through a hidden Markov random field model and the expectation-maximization algorithm. IEEE Trans. Med. Imaging *20*, 45–57.

S13. Amunts, K., Schleicher, A., Burgel, U., Mohlberg, H., Uylings, H.B., and Zilles, K. (1999). Broca's region revisited: cytoarchitecture and intersubject variability. J. Comp. Neurol. *412*, 319–341.

S14. Tomaiuolo, F., MacDonald, J.D., Caramanos, Z., Posner, G., Chiavaras, M., Evans, A.C., and Petrides, M. (1999). Morphology, morphometry and probability mapping of the pars opercularis of the frontal gyrus: An in vivo MRI analysis. Eur. J. Neurosci. *11*, 3033–3046.

S15. Devlin, J.T., and Poldrack, R.A. (2007). In praise of tedious anatomy. Neuroimage, in press.

S16. Shattuck, D.W., Hojatkashani, C., Mirza, M., Adisetiyo, V., Salamon, G., Narr, K.L., Poldrack, R.A., Bilder, R.M., and Toga, A.W. (2006). Construction of a 3D Probabilistic Atlas of Human Brain Structures. In 12th Annual Meeting of the Organization for Human Brain Mapping. (Florence, Italy).
